# Characterization and recycling of textile sludge for energy-efficient brick production in Ethiopia

**DOI:** 10.1007/s11356-020-11878-7

**Published:** 2021-01-02

**Authors:** Dawit Alemu Beshah, Girum Ayalneh Tiruye, Yedilfana Setarge Mekonnen

**Affiliations:** 1 Center for Environmental Science, College of Natural and Computational Sciences, Addis Ababa University, P. O. Box 1176, Addis Ababa, Ethiopia; 2 Ethiopia Chemical and Construction Inputs Industry Development Institute, P. O. Box 6945, Addis Ababa, Ethiopia; 3 Materials Science Program/Department of Chemistry, College of Natural and Computational Sciences, Addis Ababa University, P. O. Box 33658, Addis Ababa, Ethiopia

**Keywords:** Fired clay bricks, Zero liquid discharge, Textile industry sludge, Recycle, Energy efficiency

## Abstract

In recent years, an enormous amount of sludge is generated every day from zero liquid discharge treatment plant due to rapid expansion of industrial parks in Ethiopia. About 30,000 tons of partially dried sludge discharged to the environmental without proper waste management from all industrial parks. Thus, posing serious environmental problems. One of the most plausible means to recycle the excess sludge resource is converting it into energy-efficient brick by combining with clay. Bricks were prepared by incorporating textile sludge at different proportions (10–40%) and temperature (600, 900 and 1200 °C). Clay and sludge samples were collected from the Addis Ababa brick factory PLC and Hawassa Industrial Park. Results revealed that 10 and 20% sludge bricks satisfied criteria of class “A” bricks as per Ethiopia standards, with the compressive strength of 30.43 and 29.10 Mpa, respectively, at 1200 °C. About 26 and 50% of energy were saved during firing of 10 and 20% sludge-containing bricks, respectively, compared with pristine clay bricks. Moreover, too low concentrations of selected heavy metals found in the brick leachate, showing the sludge, were effectively stabilized in the burnt clay bricks. Thus, based on the results, we suggest the rapid utilization of huge amount of partially dried sludge resources for low-cost and efficient large-scale brick production. This will mutually benefit both the industrial parks and brick production industries. In addition, this will create thousands of jobs to the local people. Above all, the solid waste will be managed properly at textile industrial parks.

## Introduction

Textile industry sludge is one of the main pollution problems worldwide due to its dye-containing wastewater. Up to 10-25% of textile dyes is discharging to the environment during the dyeing process, and of which, 2-20% is directly released as aqueous effluents in different environmental components. Without adequate treatment, the waste-containing dyes can remain in the environment for a long period of time (Amsayazhi and Mohan 2018; Delelegn 2018; Keerthana et al. 2019; Kumar et al. 2019; Mary Lissy and Sreeja 2014; Oladejo et al. 2019; Palanisamy 2011). Most industries in Ethiopia accumulate or dispose untreated sludge on unsecure open landfills which aggravate environmental pollution. The raw sludge, coming from textile effluent treatment plant and containing heavy metals, is very active to circulate in the environment and causes environmental pollution. In addition, leaching of pollutants, especially toxic metals in the untreated textile sludge, may lead to human and animal health risks. Unlike wastewater and air emission standards, much attention is not given to land and water disposal of sludge in Ethiopia. Little report is available in Ethiopia about the presence and content of physical and chemical residues from wastewater treatment plant sludge and its recovery options (Delelegn 2018).

Accumulation of toxic metals in agricultural land during wastewater irrigation and sludge disposal will not only contaminate soil but also affect food quality and safety (Baawain et al. 2014). Therefore, the partially dried sludge must be treated or recycled before it is discharged to the environment. Most of the time, sludge is treated thermally to recover energy from it. However, the thermal treatment of sludge involves incineration, gasification, and pyrolysis as a means of disposal, which are costly and may contribute to air pollution, and the residue with high content of toxic metals still has to be disposed off to the environment (Đurđevic et al. 2019; Juel et al. 2017; Oladejo et al. 2019). An alternative technique to treat or stabilize hazardous waste is solidification method and using the solid as construction materials such as ground leveling, brick, or concrete. These can be applied in several instances for the cases of sewage, textile sludge, and arsenic-rich filter materials (Arsenovic et al. 2012; Barnat-hunek and Wdowin 2016; Patel and Pandey 2012; UNDP et al. 1998). Moreover, sewage sludge ash can also provide as resource for phosphorous and material for clay brick manufacturing (Ottosen et al. 2020).

Stabilization of hazardous waste usually refers to a technique that reduces the toxicity of a waste by converting the toxic substances into less soluble or mobile form. Solidification also refers to the physical phenomenon that solidifies the waste, forming solid materials, and does not necessarily involve a chemical interaction between the contaminants and the solidifying additives (Arsenovic et al. 2012; Hassan et al. 2014; Patel and Pandey 2012, 2009; UNDP et al. 1998). The solidified product may be disposed off to a protected landfill site, or it can be recycled as construction materials if it satisfies the specific strength requirement and contains toxic pollutants within acceptable limits. Several previous studies demonstrated that textile industry sludge can be combined with clay material to form energy-efficient bricks and effectively stabilized hazardous wastes (Aouba et al. 2016; Hassan et al. 2014; Mary Lissy et al. 2018; Patel and Pandey 2009; Ravikrishnan and Senthilselvan 2014; Weng et al. 2003). In addition to textile sludge, utilization of different waste sources were investigated as brick materials such as water treatment (Niwagaba et al. 2019)(Algamal et al. 2018), tannery (Amsayazhi and Mohan 2018) (Juel et al. 2017), iron and steel making (Hassan et al. 2014; Vieira et al. 2018), effluent from paper industry (Bhushan et al. 2019; Sarkar et al. 2017), stockpiles (Bricks et al. 2019), and waste from rice husk (Hegazy et al. 2012). Moreover, the reuse of waste clay bricks from old buildings (Cheng 2016) and fly ash (Kadir and Sarani 2012) can also be used as a raw material for brick-making.

The Ethiopian Industrial Park Development Corporation (IPDC) has constructed about 13 industrial parks so far, and other 10 industry parks are planned and started constructing throughout the country. It is estimated that about tons of partially dried sludge will be generated each year by all the industrial parks if they become fully operational, indicating the necessity of management of the produced sludge before it is disposed off Moreover, large- and small-scale brick manufacturing industries in Ethiopia are using non-renewable energy sources for firing of the clay bricks.

For this study, partially dried sludge was collected from one of the industrial parks in Ethiopia called Hawassa Textile and Apparel Industrial Parks, which is located 275 km from Addis Ababa*.* The main aim of this study was to characterize the collected textile sludge and to convert it into energy-efficient bricks by combining with clay. In addition to suitably managing huge amounts of industrial parks waste, brick manufacturing industries can also be significantly benefited by recycling textile sludge and contribute to produce energy-efficient bricks by combining with clay, thus minimizing the amount of energy needed to produce bricks, consequently reducing the production cost. This can be one of the most practicable options for marketable application because of the mineralogical property and calorific value of the sludge.

## Materials and methods

### Sample preparation and characterization

#### Sample preparation

**Fig. 1:**
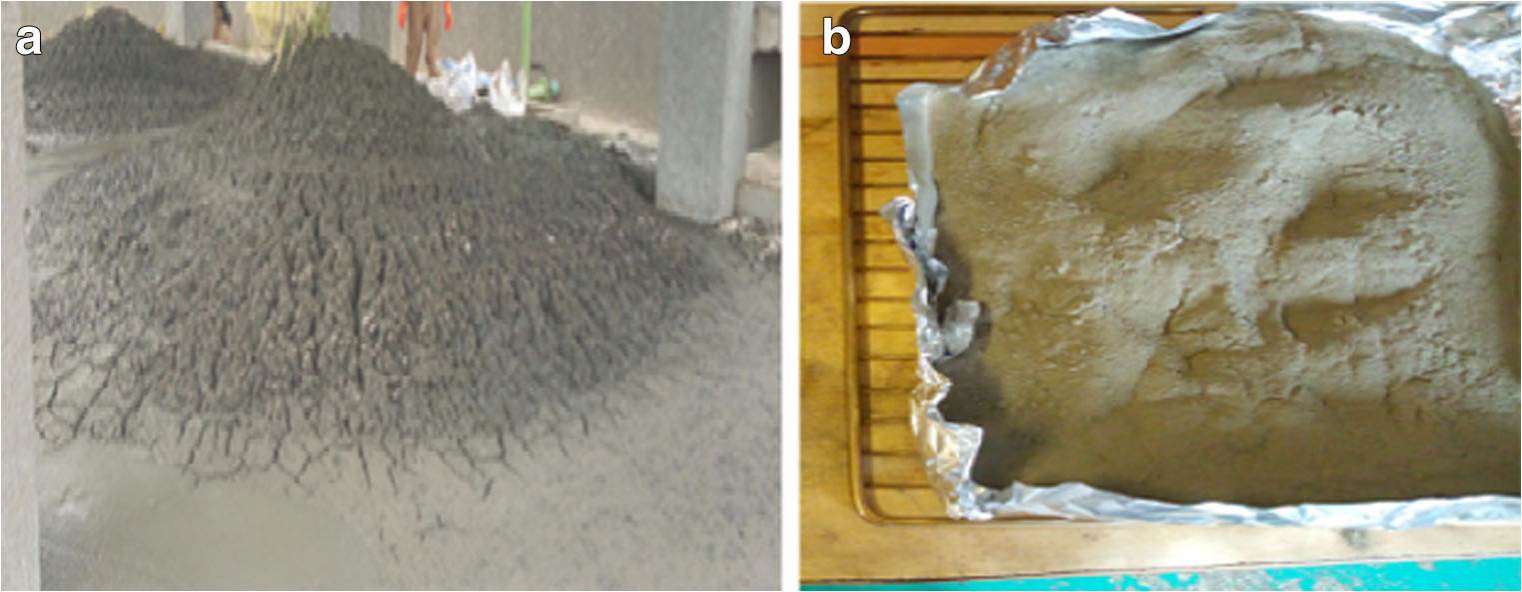
**a** Textile sludge and **b** pulverized textile sludge

Clay sample was collected from Addis Ababa Brick Factory, which is a private limited company (PLC) located in Addis Ababa, Ethiopia. The collected clay samples were air dried and then used for preparing bricks by combining with textile sludge. Textile sludge was collected from a zero liquid discharge treatment plant from Hawassa Textile Industry Park, from which about 80 tons of textile sludge is generating daily. The zero-water discharge treatment plant sludge was air dried and sieved for removal of dusts. It was then pulverized to powder form using a soil grinder and passed through 63-μm standard sieve; see Fig. 1. The sludge sample became ready to mix with the clay at various proportions to form bricks. The textile sludge has a high calcium and magnesium content, which comes mainly from coagulating chemicals used in the zero liquid discharge treatment plant.

#### Characterization of sludge and clay samples

The physicochemical characteristics of each sample were determined. The pH of each sample was determined following the USEPA standard method (USEPA 2004). The ash, moisture, and organic contents in terms of percentage were obtained using the ASTM D 2974-87 method (ASTM D 2974-87 1993). Wet method was followed to determine the moisture content of samples. Sludge samples of 2.5 g were taken in triplicate and dried at ambient temperature. Thereafter, the samples were dried in oven at 105 °C for about 24 hours, followed by cooling in the desiccator for 30 minutes Then the final weight of the cooled samples were measured again. Clay samples were prepared from the mixture of 25% red and 75% white soil samples as the brick manufacturing used this composition for typical brick production.

The morphology and chemical composition of textile sludge and clay samples were identified using X-ray diffraction spectroscopy (XRD) and scanning electron microscopy coupled with energy dispersive X-ray spectroscopy (SEM-EDX) techniques using Mini Flex 600 XRD and JSM-IT300 SEM-EDX, respectively. Differential scanning calorimetry (DSC) tied with thermogravimetric analysis (TGA) of textile sludge sample was examined using SDT Q600 V20.9 Build 20 instrument.

### Brick preparation and characterization

**Fig. 2:**
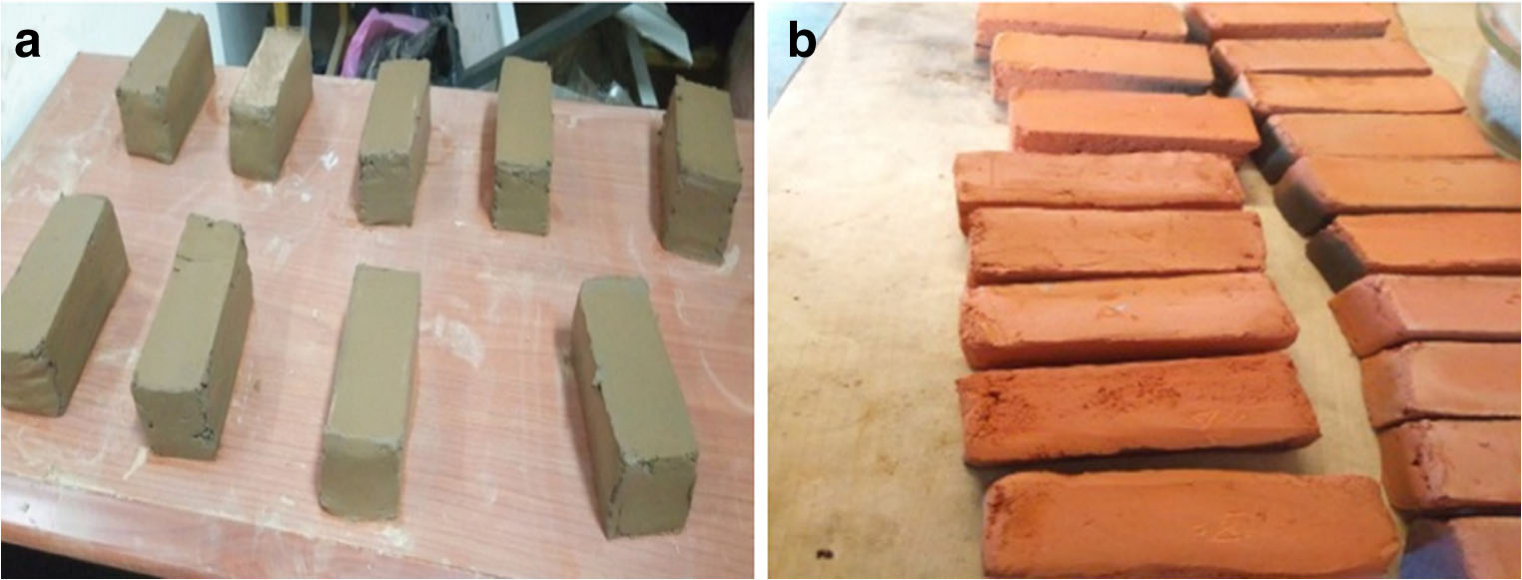
**a** Oven-dried bricks and **b** fired bricks

The clay and sludge samples were oven-dried and grounded using a crushing machine. Total of 135 bricks sample (length, 120 mm; width, 50 mm; and height, 50 mm) of sludge-clay mixture in varying proportions (10:90, 20:80, 30:70, and 40:60 by w/w) were prepared in the laboratory. Additional 4% water was added to all the sample mixed ratios to make the hand molding process while mixing easy. Three control clay samples were prepared as a reference. After 24 hours of air drying in open area, 48 hours of oven-drying at 105 °C (Fig. 2a) was carried out. The prepared bricks were subjected to heating at 600, 900, and 1200 °C for 6 hours with furnace (SX-2.3-10 Muffle Furnace) at the rate of 5 °C/min (Fig. 2b).

Physicochemical characteristics of the bricks such as percentage of water absorption, weight loss on ignition, firing shrinkage, and compressive strength were performed as per the ASTM C67-09 standard test methods (ASTM C67-09 2009). Moreover, heavy metal leachability test was conducted for all brick samples in line with USEPA 1311 (USEPA 1992). The test results of all parameters were taken as the mean value of three replicate tests.

#### Water absorption of bricks

The water absorption of the brick samples was conducted according to ASTM C67-09 testing method (ASTM C67-09 2009). Based on this method, the brick samples were oven-dried at 105 °C for 24 hours. After drying, the samples were cooled at room temperature. The dried and cooled brick was submerged, without preliminary partial immersion, in distilled water at 27 °C for 24 hours. Then the brick was wiped off the surface water with a damp cloth, and the weight of the brick was taken within 5 minutes after removing the brick from the bath. Water absorption of the sample was calculated using Eq. 1.

1 $$ \mathrm{Water}\ \mathrm{absorption}\%=\frac{\ \left({W}_{\mathrm{s}}-{W}_{\mathrm{d}}\right)}{W_{\mathrm{d}}}\times 100 $$

where *W*_d_ refers to the dry weight of the specimen and *W*_s_ is the saturated weight of the specimen after submersion in cold water.

#### Weight loss on ignition

The brick samples were oven-dried at 105 °C for 24 hours. Then the dried bricks were fired at 600, 900, and 1200 °C. Loss of Ignition (LOI) was determined by measuring the weight loss of the sample between the drying and firing stages as described in Eq. 2.

2 $$ \mathrm{LOI}\%=\frac{\left({W}_{\mathrm{d}}-{W}_{\mathrm{f}}\right)}{W_{\mathrm{d}}}\times 100 $$

where *W*_d_ is the mass of oven-dried specimens (g), and *W*_f_ is the mass of fired specimens (g).

### Firing energy saving

Brick production industries are energy-intensive sectors and have a large negative impact on the environment relating to energy consumption (Koroneos and Dompros 2007). Saving energy consumption is of high importance in the brick manufacturing industry and can be done by incorporating sludge to the clay, resulting in a sustainable brick production. The energy saved due to incorporating sludge can be calculated from the equation described in the previous work (Mohajerani et al. 2016). The textile sludge used in this study contains about 20.7% organic matter which could facilitate heat input to the furnace and reduce the amount of energy required for firing. The amount of energy saved from each brick sample was calculated from specific energy of bricks and mass of clay and sludge in control and sludge bricks as shown in Eq. 3.

3 $$ \mathrm{Energy}\ \mathrm{saved}=\frac{q{m}_1-\left(q{m}_2-{C}_{\mathrm{v}}{M}_3\right)\times 100}{q{m}_1} $$

where *q is* the specific energy for brick-making through firing, and the specific energy for the usual brick-making is assumed to be 3 MJ/kg. *m*_1_ is the mass of clay in control brick (kg), *m*_2_ is the mass of sludge brick (kg), M_3_ is the mass of sludge in sludge brick (kg), and *C*_V_ is calorific value of textile sludge (MJ/kg). The specific firing energy was measured by dividing the firing energy (MJ) with the mass of brick (kg).

### Leaching test of bricks

The leachability tests were carried out at different sludge-to-clay proportions to ensure the environmental compatibility of these fired bricks to be used as building materials. In this study, the toxicity characteristic leaching procedure (TCLP) test was carried out according to USEPA Method 1311 (USEPA 1992). In the TCLP test, dried brick samples which were fired at 1200 °C were grounded using a grinder and passed through 9.5-mm standard sieve. An acetic acid solution (0.57% v/v) was added to samples at a constant ratio of liquid:solid (20:1 w/w). After 18 hours rotating with a rotary mixture at 3 ± 2 rpm, the leachate was filtered with 0.45-μ pore size filter paper and analyzed for Cu, Ni, Pb, and Zn using flame atomic absorption spectroscopy (FLAAS) at the Geological Survey of Ethiopia.

## Results and discussion

### Characteristics of the sludge and clay samples

**Fig. 3:**
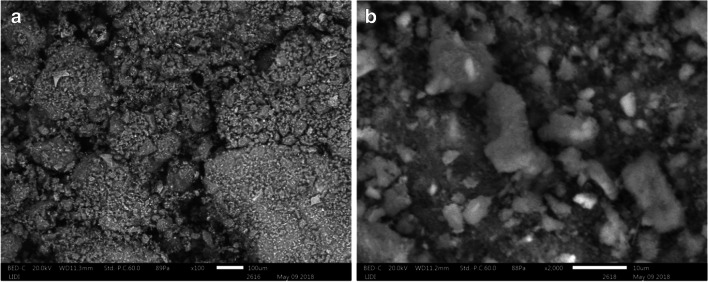
SEM images of textile sludge: **a** ×100 μm **and b** ×10 μm magnification

The morphological feature of the textile sludge was characterized by the scanning electron microscope (SEM) imaging technique. As it can be seen in Fig. 3, rough, agglomerated, and cracked surface of textile sludge indicates a heterogeneous and amorphous structures of particles.

The textile sludge and clay were also characterized by using various physicochemical methods before processing the brick production. Table 1 shows the pH, moisture content, organic content, and metals content (mg/kg) for the textile sludge and clay. The moisture content of the textile sludge (21.4%) was found to be significantly higher than that of the clay sample (4.9%). Similarly, the organic carbon content of textile sludge and clay were found to be about 20.7% and 3.6 %, respectively.

**Table 1:** Physicochemical characteristics of the sludge and clay sample.

Properties	Sludge	Clay
pH	7.44	6.31
Moisture content (%)	21.4	4.9
Total organic carbon (%)	20.7	3.6
Net calorific value (MJ/kg)	4.65	-
Ash content (%)	43.3	-

The textile sludge composition can vary from company to company depending on the textile manufacturing processes applied and chemicals involved for treatment. As shown in Table 2, the main components of the textile sludge ash contents used in this study were characterized and found to include the following combination: Al_2_O_3_ (36.2%), SiO_2_ (15%), SO_3_ (13.40%), Fe_2_O_3_ (12.10%), CaO (3.50%), MgO (2.80%), and other trace compounds.

**Table 2:** Chemical composition and phase of sludge and clay soil characterized by XRD analysis

Analytes	Sludge (%)	Clay soil	
		White soil (%)	Red soil (%)
SiO_2_	15.00	66.50	55.30
CaO	3.50	< 0.01	< 0.01
Al_2_O_3_	36.20	11.36	16.40
Fe_2_O_3_	12.10	8.16	9.62
SO_3_	13.40	-	-
MgO	2.80	0.16	0.60

**Fig. 4:**
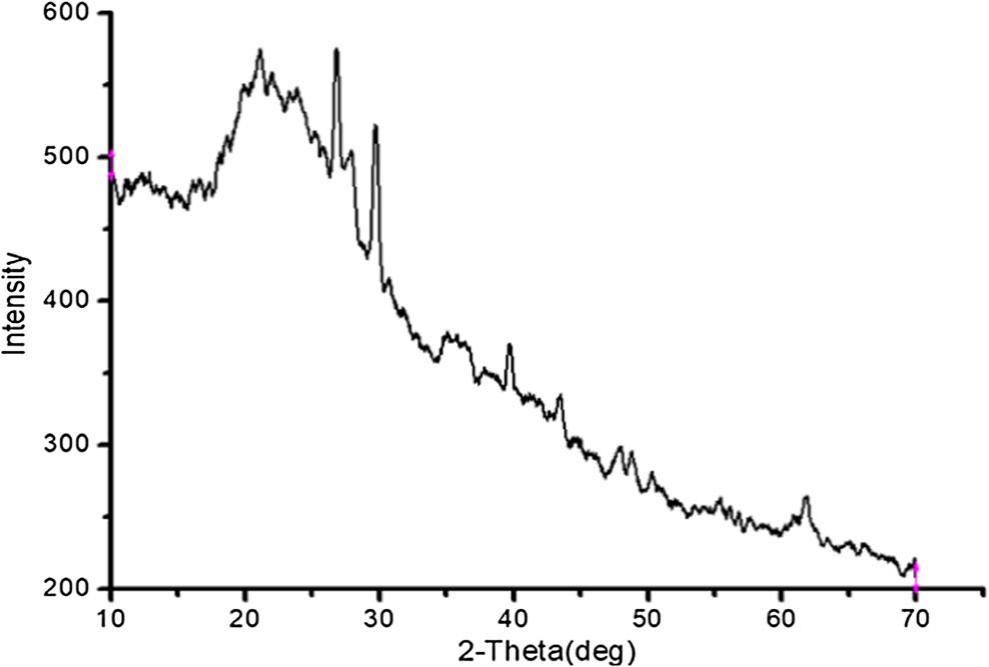
XRD pattern for the sludge sample

The clay that was used for brick manufacturing consists of similar components present in the textile sludge but with different quantities. So, clay can be partially replaced by textile sludge as raw material for large-scale brick production. Katte et al. (2017) have reported the following oxides as major components of the clay sample in Cameroon: SiO_2_ (54.9%), Al_2_O_3_ (23.4%), Fe_2_O_3_ (4.8%), MgO (0.9%), CaO (0.5%), and Na_2_O (0.24%) (Katte et al. 2017). Similarly, Barnat-hunek and Wdowin (2016) have also confirmed the presence of high content of SiO_2_, Al_2_O_3_, Fe_2_O_3_, and K_2_O in a clay sample (Barnat-hunek and Wdowin 2016). The XRD measurement showed that the textile sludge is mainly an amorphous structure as shown Fig. 4.

Likewise, the XRD result analysis shows that the main chemical composition of the clay (white and red soils) sample was found to be predominantly composed of the subsequent oxides SiO_2_ (66.5 and 55.3%), Al_2_O_3_ (11.36 and 16.4%), Fe_2_O_3_ (8.16 and 9.62%), and MgO (0.16 and 0.6%) (Table 2).

**Fig. 5:**
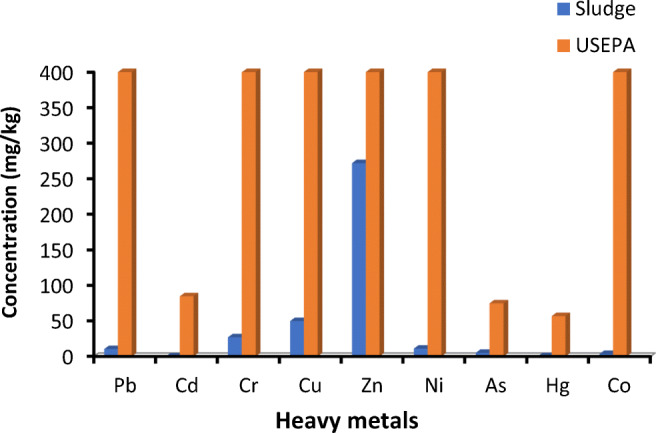
Heavy metal concentration (mg/kg) in the sludge analyzed by FLAAS compared with USEPA restriction limit

The textile sludge was found to contain zinc metal as the principal component, and other heavy metals present in the sludge were to be found in decreasing order as Zn > Cu > Cr > Ni > Pb > As > Co > Hg > Cd**.** The mean concentration (mg/kg) of each heavy metal in sludge was found to be Zn (272), Cu (50), Cr (27), Ni (11), Pb (10), As (5), Co (3.3), Hg (0.4), and Cd (0.26). As it can be seen in Fig. 5, the concentration of all heavy metals present in the textile sludge were found to be far below the values of USEPA standard for land application and land disposal restrictions of sludge (USEPA 1994)**.**

The EDX analysis performed to establish the elemental composition of the textile sludge sample. As shown in Table 3, the key elemental compositions of the sludge sample were O, C, Al, and Si. These components are also likewise found in clay sample. Since most of the elemental composition and oxides of sludge are comparable with clay material, the latter can be partially replaced by textile sludge as raw material for brick production (Wiemes et al. 2017; Junaid et al. 2018; Coletti et al. 2016).

**Table 3:** Elemental composition of the sludge sample obtained from EDX analysis

Element	C	N	O	Na	Mg	Al	Si	S	K	Ca	Fe	Total
Mass (%)	18.8	7.9	48.7	0.7	0.8	13	6.7	1.1	0.3	1.2	0.8	100

**Fig. 6:**
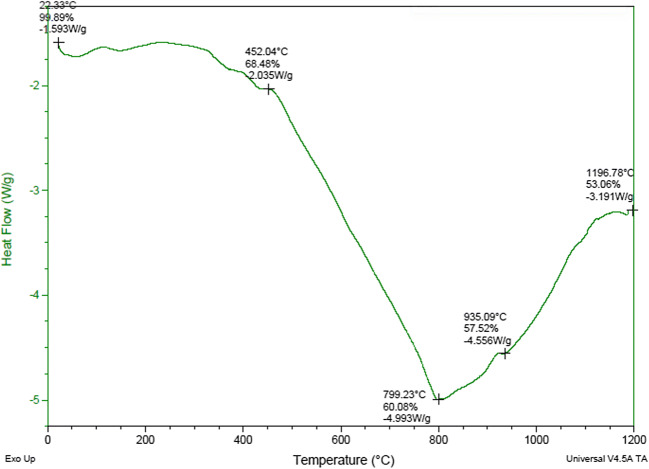
DSC-TGA plot of textile sludge sample

Differential scanning calorimetry (DSC) and thermogravimetric analysis (TGA) of sludge provide information on thermal behavior of the sludge. The plot on Fig. 6 shows three distinct heat flow patterns, which correspond to gradual and step weight loss variations at different temperature ranges. In the temperature interval range from 452 to 800 °C, there was a gradual decrease in weight mainly due to loss of water and organic matter. A steep weight loss (about 8% mass loss) and heat flow around 5 W/g were observed between 452.04 and 799.23 °C temperature interval, which may be mainly due to melting of sludge and form liquid phase. The curve then shoots up on the way until 1196.78 °C, where the rate of weight loss was about 3 W/g, wherein recrystallization is carried out.

### Properties of fired bricks

#### Compressive strength of bricks

**Fig. 7:**
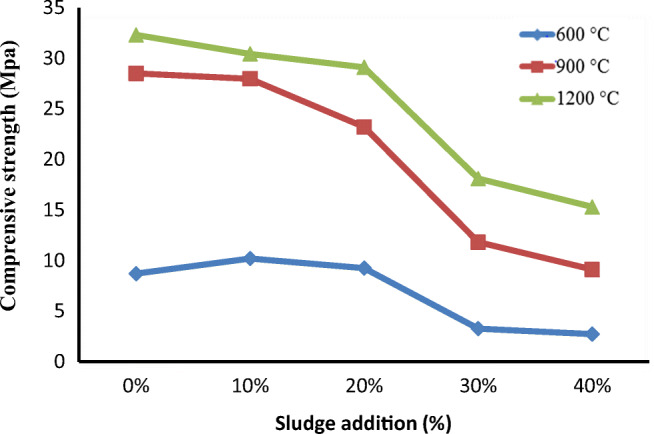
Compressive strength of the brick samples at different temperature

For confirming, the engineering quality of a building material compressive strength test is the key parameter to evaluate the strength of the bricks. The compressive strength of textile sludge incorporated bricks ranging from 2.73 to 30.43 Mpa. It was observed that the content of sludge and firing temperature have a significant effect on the compressive strength of bricks. As shown in Fig. 7, it was revealed that the compression strength increased with the firing temperature, probably due to the burning off organic components and compacting the bricks. This was confirmed by increasing the sludge content in the brick composition, which results in inversely related sludge content and compression strength. The compression strength of the bricks was decreased with increasing the sludge content. The compression strength of 10 and 20% sludge substituted bricks prepared at 900 and 1200 °C satisfied the requirements of class “A” brick as per the Ethiopian conformity assessment enterprise brick standards (ESA 2011).

#### Water absorption of bricks

**Fig. 8:**
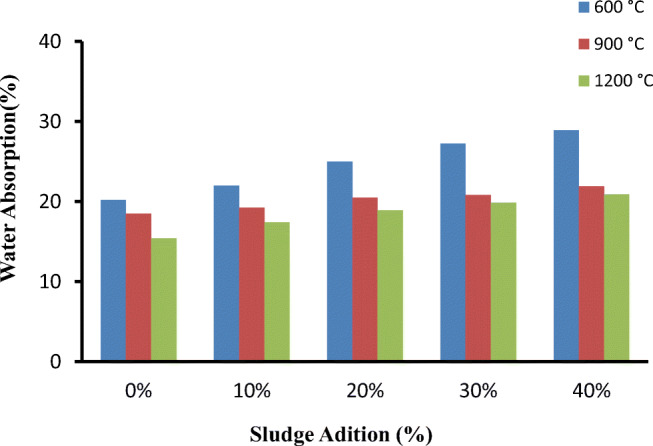
Water absorption results of bricks in percent at different temperature

The less water penetrates into the brick, the more durability of the brick and resistance to harsh weather conditions (Hegazy et al. 2012). Thus, the internal structure of the brick must be compact enough to avoid the intrusion of water. It has been found that the water absorption of the bricks increased with increased textile sludge, indicating potential increase in its susceptibility to weathering action. When the brick with 100% clay (0% sludge content) was fired at 600 °C, the water absorption was found to be 20%. This water absorption was increased by 50% from that of the 100% clay brick when the sludge content was increased by 40% and fired at 600 °C. Generally, textile sludge contains a high amount of organic matter which is responsible for cavities created in the brick during firing, and these cavities favor water absorption. The textile sludge used in these experiments contained a high amount of organic content (20.7%), and it was found that the quantity of absorbed water increased with the increase of textile sludge proportion (Fig. 8). In contrast, the water absorption of brick was decreased with increasing the firing temperature, attributing to the amorphous phase formation during firing at high temperature. As can be seen in Fig. 8, a brick containing 10% textile sludge burnt at 600 °C exhibited water absorption of 22%, which was reduced to 17.4% when firing temperature increased to 1200 °C. Likewise, some previous studies have also reported similar trends (Arsenovic et al. 2012; Katte et al. 2017; Ramadan et al. 2008; Tay 1987)

#### Weight loss on ignition

**Fig. 9:**
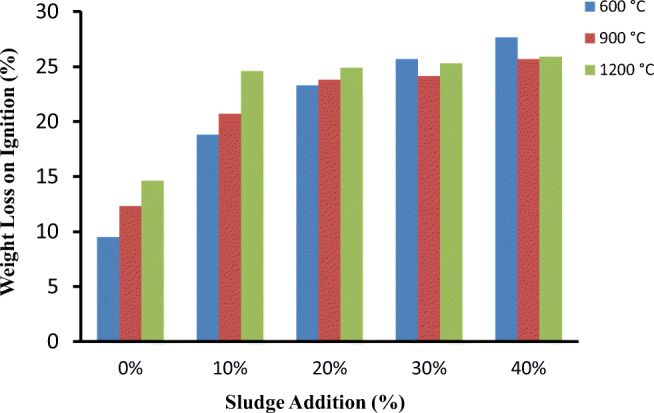
Weight loss on ignition in bricks of different sludge percentage.

The weight loss on ignition depends on both organic matter content and inorganic substance that are found in both clay and sludge being burnt off during the firing process (Baskar et al. 2006; Sulthana and Gandhimathi 2013) . The effect of sludge content and firing temperature on weight loss of bricks are shown in Fig. 9. Loss of ignition (LOI) was found to be high at higher sludge content. For the bricks fired at 1200 °C, the loss on ignition was 24.75% for 30% sludge bricks. The weight loss on ignition has a linear relationship with the sludge content; as the sludge content increases from 10 to 40%, the weight loss on ignition has increased from 24.6 to 25.9%, respectively. As the percentage of sludge used for the brick increases, the final weight of the brick reduces. This can be considered a positive result, due to decreasing the overall dead weight of the masonry, and a lower strength brick may be required. An overall reduction in weight may also have positive effects on transport costs as these are often calculated by weight. In addition, as the structure is lighter in weight, smaller foundations may be required leading to further overall cost reductions.

This weight loss could be due to the combustion and decomposition of the organic and inorganic matter present in both textile sludge and clay during the firing at high temperature. Similar kinds of weight loss results were reported in case of sewage sludge and textile sludge incorporated bricks in other studies (Ottosen et al. 2020)(Jahagirdar et al. 2013).

### Firing energy saving

The incorporation of textile sludge in bricks has the potential to reduce the energy required during firing. Currently, energy efficiency and environmental concerns have become as great an issue as quality and cost in the manufacturing sector, due to increased awareness of the effects on the environment (Mohajerani et al. 2016). Calorific value of textile sludge, which was measured by a bomb calorimeter, was found to be 4.63 MJ/kg. The estimated amount of energy saved during firing of textile sludge bricks was calculated using Eq. 3. Despite the decrease in compression strength up on increasing the sludge content, bricks with higher sludge can efficiently save the energy consumption. About 26% energy would be saved for 10% textile sludge bricks and 50% energy saving for 20% textile sludge bricks during firing compared with control bricks at a firing temperature of 1200 °C. A similar result of energy saving was reported for ETP biosolid–incorporated bricks and for cigarette butt–incorporated bricks (Mohajerani et al. 2016). Therefore, textile sludge used in this study contained about 20.7% organic content which could facilitate heat input to the furnace and reduce the amount of energy required for firing. Previous studies conducted in Asia have shown that the specific firing energy consumption per a brick was approximately between 2 and 3 MJ/ kg (Prasertsan 1995; Prasertsan and Theppaya 1995), whereas for organic matter content in the biosolids the calorific value can reach up to 22 MJ/kg (Oladejo et al. 2019). For this study, the specific energy consumption is found to be 3 MJ/kg.

### Leaching test of the bricks

The leaching test for some selected toxic heavy metal concentration was designed to identify hazardous wastes that can potentially leak from leachate into the ground water. During the hazardous metals test, constituents are extracted from the waste to simulate leaching actions that actually occur in landfills. If the concentration of the toxic constituents exceeds the regulatory limit, the waste is classified as hazardous. The leachate analysis was characterized according to USEPA 1311 (USEPA 1992). Pb almost did not leach out from the sludge-amended bricks fired at 1200 °C as their concentrations were far below the detection limit of the instrument. Moreover, as shown in Table 4, other heavy metals, namely, Co, Cu, and Zn leached out from sludge-amended brick concentration were found to be far below the USEPA regulatory limits that are 4.0, 6.8, and 12.2 ppm, respectively. Thus, the sludge was effectively stabilized by mixing with clay in the process of brick production at high temperature.

**Table 4:** Leaching test of 10% sludge brick fired at a temperature of 1200 °C

	Pb	Co	Cu	Zn
Concentration of raw sludge (mg/kg)	10.4	3.3	50	272
Concentration of leached out from brick sample (mg/kg)	< 0.01	4.0	6.8	12.2

## Conclusions

In conclusion, energy-efficient bricks were prepared by incorporating textile sludge at different proportions and temperatures. The sludge characterization analysis revealed that the heavy metal content of the sludge was found to be very far below USEPA regulatory limit. All sludge bricks produced at 900 and 1200 °C were found to satisfy the requirements for class “A” brick category of Ethiopian standards. The compressive strength of textile sludge bricks reduced considerably from 30.43 to 2 MPa when the textile sludge content increased from 10 to 40%, and it increases with firing temperature. As the sludge content in the brick increase, the amount of energy saved in the production increases. It was evaluated that energy can be saved up to 26 and 50% for 10 and 20% sludge, respectively. A 10% sludge brick suitably satisfies the standards of good-quality brick with the compressive strength of 30.43 MPa at 1200 °C, which is highly comparable with pure burnt clay bricks (32.3 MPa) at the same temperature. An increase in both the content of sludge and the firing temperature caused higher weight loss in the manufactured bricks. This study showed that at 10 and 20%, sludge mixed with clay burnt at a temperature of 900 and 1200 °C can possibly produce cost-effective and good-quality bricks that can satisfy all the necessary brick characteristics in accordance with the standards. In order to properly manage the huge amount of sludge generated every day from textile industrial parks in Ethiopia, converting the sludge into energy efficient bricks can pave the way for recycling the sludge into value-added bricks for various purposes, hence creating economic fortunes to the brick manufacturing industries as it saved energy consumption (saving money) and decreasing the final weight of the brick which decreases the transportation cost.

## Data Availability

All data generated or analyzed during this study are included in this published article.
